# TrkA serves as a virulence modulator in *Porphyromonas gingivalis* by maintaining heme acquisition and pathogenesis

**DOI:** 10.3389/fcimb.2022.1012316

**Published:** 2022-11-02

**Authors:** Renjie Zou, Lei Zhao, Daonan Shen, Yafei Wu

**Affiliations:** State Key Laboratory of Oral Diseases, National Clinical Research Center for Oral Diseases, West China Hospital of Stomatology, Department of Periodontics, Sichuan University, Chengdu, China

**Keywords:** potassium ion uptake regulatory protein, *Porphyromonas gingivalis*, high-throughput sequencing, heme acquisition, pathogenicity

## Abstract

Periodontitis is an inflammatory disease of the supporting tissues of the teeth, with polymicrobial infection serving as the major pathogenic factor. As a periodontitis-related keystone pathogen, *Porphyromonas gingivalis* can orchestrate polymicrobial biofilm skewing into dysbiosis. Some metatranscriptomic studies have suggested that modulation of potassium ion uptake might serve as a signal enhancing microbiota nososymbiocity and periodontitis progression. Although the relationship between potassium transport and virulence has been elucidated in some bacteria, less is mentioned about the periodontitis-related pathogen. Herein, we centered on the virulence modulation potential of TrkA, the potassium uptake regulatory protein of *P. gingivalis*, and uncovered TrkA as the modulator in the heme acquisition process and in maintaining optimal pathogenicity in an experimental murine model of periodontitis. Hemagglutination and hemolytic activities were attenuated in the case of *trkA* gene loss, and the entire transcriptomic profiling revealed that the *trkA* gene can control the expression of genes in relation to electron transport chain activity and translation, as well as some transcriptional factors, including *cdhR*, the regulator of the heme uptake system hmuYR. Collectively, these results link the heme acquisition process to the potassium transporter, providing new insights into the role of potassium ion in *P. gingivalis* pathogenesis.

## Introduction

*Porphyromonas gingivalis* is a gram-negative bacteria considered as the keystone pathogen of periodontitis. While coinhabiting with various microorganisms in the subgingival pocket, it can orchestrate the whole virulence of the community, named nososymbiocity, regardless of its relative minor biomass (Hajishengallis et al., 2011). Equipped with miscellaneous virulence factors including but not limited to gingipains, fimbriae, and untypical lipopolysaccharide, *P. gingivalis* can subvert the host response by paralyzing the function of complement and immune cells while accentuating the hyperinflammatory state, such that more pathobionts will multiply synergistically for nutrition acquisition and persistence in the inflammatory environment which certainly creates a skewing to dysbiosis of subgingival flora (Hajishengallis et al., 2012).

Microbial dysbiosis is the initial factor of periodontitis. Following longitudinal monitoring of periodontal attachment level, Socransky and his colleagues put forward a model of periodontitis in which attachment loss undergoes acute bursts for short periods randomly in individual sites (Socransky et al., 1984). Some clinical trials have attempted to associate periodontitis progression with dysbiosis features, whereas most studies reported an overlap in the constitution of the microbial community during different phases of progression (Teles et al., 2008; Byrne et al., 2009). Remarkably, a recent omics study on metatranscriptomic analysis of subgingival plaque biofilm suggested a link of cobalamin biosynthesis, proteolysis, and potassium transport activity to periodontitis progression (Yost et al., 2015). Subsequent research confirmed that potassium ion was associated with enhanced virulence of the plaque community *in vitro* (Yost et al., 2017). As the keystone pathogen of periodontitis, it is thus of interest to determine what function alterations and underlying mechanisms of *P. gingivalis* would be brought about when the level of potassium ion or corresponding transporter activity was manipulated.

The virulence potential of potassium cation and transporter has been investigated in some pathogens in the field of environmental cues and host cell colonization. Potassium transporter is indispensable for *Salmonella enterica* serovar Typhimurium to express type III secretion (T3SS) and conduct swift intestinal invasion (Liu et al., 2013). The secretion of virulent factors by T3SS can be also affected by a high concentration of intracellular potassium ion (Tandhavanant et al., 2018). The studies on the inactivation of the potassium uptake system in *Vibrio vulnificus* (Carda-Dieguez et al., 2018), *Francisella tularensis* (Alkhuder et al., 2010), and *Mycobacterium tuberculosis* (MacGilvary et al., 2019) have also highlighted the link with host defense resistance and colonization. Beyond bacteria, potassium still works as the signal for the parasite *Plasmodium* spp. egress from the host cells and bunyavirus genome release during endosomal trafficking (Moraes et al., 2017; Hover et al., 2018). Those phenomena mark potassium ion as an extensive signal for bacteria to show virulence.

While the impact of disruptive potassium homeostasis on bacterial pathological roles is beginning to be elucidated, little is known about *P. gingivalis* as well as the underlying effect of potassium in linking *P. gingivalis* to periodontitis. Herein, we attempted to decipher the role of TrkA, the regulatory subunit of the potassium uptake system Trk in *P. gingivalis* W83. To achieve that, the *trkA* mutant was constructed, and we undertook sequential investigations. Finally, our data associated the function of the *trkA* gene with the heme acquisition process as well as virulence in the murine experimental periodontitis model, which laid the basis for elucidating the role of potassium ion in periodontitis progression.

## Material and methods

### Bacterial culture

*Porphyromonas gingivalis* W83 strains were cultured in an anaerobic chamber at 37°C in tryptic soy broth (TSB) medium supplemented with 5 μg/ml of heme and 1 μg/ml of menadione. For the culture of its isogenic *trkA* mutants and complemented strains, erythromycin or tetracycline at a concentration of 10 or 0.6 μg/ml was added to the medium. *Escherichia coli* strains S17-1 were cultivated aerobically at 37°C and 200 rpm in Luria–Bertani medium added with 50 µg/ml of carbenicillin or 5 µg/ml of tetracycline whenever applicable.

### Mutant construction and complementation

For the construction of Δ*trkA* mutants, *ermF* cassette was first amplified and fused with the upstream and downstream sequences of the *trkA* gene using overlap extension PCR. The primers applied in the former procedure are shown in **Table S1**. The recombinant DNA fragments obtained were purified, sequenced, and subsequently electroporated into electrocompetent *P. gingivalis* cells. After a 10-day anaerobic incubation on TSB plates supplemented with erythromycin, the positive clones were selected and confirmed through colony PCR analysis.

To generate the complemented Δ*trkA*/*trkA* strain, operon analysis was conducted to confirm the *trkA* operon structure such that recombinant pT-COW (kindly donated by Prof. Richard J. Lamont) can be generated by incorporating the encoding sequence and the promoter region of the *trkA* gene. Then, the constructs were transformed into Δ*trkA*/*trkA* cells by being conjugated with *E. coli* S17-1 carrying recombinant pT-COW. Positive transconjugants were selected with erythromycin, gentamicin, and tetracycline.

### Transcriptomic sequencing and bioinformatic analysis

The total RNAs of three biological replicates were extracted from the cells using TRIzol reagent according to the manufacturer’s instructions (Invitrogen, Carlsbad, USA), and genomic DNAs were removed by DNase (TaKaRa, Kusatsu, Japan). After depleting ribosomal RNA, RNA-seq transcriptome library was prepared using the TruSeq™ RNA sample preparation kit (Illumina, San Diego, USA). Then, the paired-end RNA-seq sequencing library was sequenced with the Illumina HiSeq×TEN (2 × 150 bp read length). Reads were mapped to the reference genome of *P. gingivalis* W83 (Acuna-Amador et al., 2018). Normalized read counts based on transcripts per million mapped reads (TPM) for the gene expression level were calculated.

All bioinformatic analyses were performed using the online platform of Majorbio Cloud Platform (http://www.majorbio.com). Specifically, trimmed read counts were used to determine differential gene expression *via* DESeq2 packages. Data processed in the form of log_2_ fold change expression with adjusted *p*-values were obtained. Then, the threshold of 1.0 for log_2_ fold change expression and adjusted *p*-values of less than 0.05 were used to determine differentially expressed genes. For gene ontology (GO) enrichment analysis, GOATOOLS (https://github.com/tanghaibao/GOatools) was used to identify statistically significantly enriched GO terms using Fisher’s exact test with the adjusted *p*-values of less than 0.05.

### Hemagglutination assay

The hemagglutination activity of *P. gingivalis* W83 was analyzed in reference to what was previously described (Fujise et al., 2017). Briefly, *P. gingivalis* W83 growing to the mid-log phase was collected and spun at 8,000 rpm for 10 min. The pellets were washed and diluted to a final OD_600_ of 1 in phosphate-buffered saline (PBS). A 2-fold diluted bacteria suspension was equally mixed with 1% sheep erythrocytes in a round-bottom 96-well plate. After incubation at 4°C for 4 h, the hemagglutination titers were compared among WT, Δ*trkA*, and Δ*trkA*/*trkA*.

### Hemolytic activity

Hemolysis assay of *P. gingivalis* W83 was conducted as previously mentioned (Vanterpool et al., 2004). Bacteria cultured in sTSB medium were harvested by centrifugation (3,000*g* for 10 min). The pellets were collected, washed, and resuspended again in PBS to the final OD_600_ of 1.5. After incubation with an equivalent volume of 1% sheep erythrocytes for 3 h at 37°C, the samples were spun at 1,300*g* for 5 min in a Heraeus Multifuge X1R centrifuge at room temperature. The optical absorbance of the supernatant at 405 nm was determined by spectrometry. The erythrocytes were used alone as the negative control. After normalized by the negative control and bacterial amount, the ratio of OD_405_ of the treatment groups to WT was calculated to determine relative hemolytic activity.

### Gingipain activity

N-Benzoyl-DL-arginine-*p*-nitroaniline hydrochloride (Macklin, Shanghai, China) and N-(p-Tosyl)-Gly-Pro-Lys 4-nitroanilide acetate salt (Sigma-Aldrich, St. Louis, USA) were utilized as the substrate for testing the arginine (Rgp)-dependent and lysine (Kgp)-dependent gingipain activity of *P. gingivalis* W83 WT, Δ*trkA*, and Δ*trkA*/*trkA* (Fujise et al., 2017). Briefly, the mid-log phase culture of *P. gingivalis* W83 was collected and centrifuged at 8,000*g* for 10 min at 4°C. The resulting pellets were then washed and resuspended in 10 mM of HEPES/NaOH as the whole cells. To prepare the reaction buffer, 0.2 mM of substrate, 50 mM of Tris–HCl (pH 8.0), and 10 mM of DTT were added. Reactions were proceeded by adding 4 μl of bacterial suspension of whole cells at 37°C for 30 min and terminated using acetate acid (50%). The release of *p*-nitroanilide from the substrate was then determined by spectrometry at 450 nm. The gingipain activity measurements of *P. gingivalis* WT, Δ*trkA*, and Δ*trkA*/*trkA* were normalized by bacterial density at 600 nm in HEPES/NaOH buffer.

### Membrane potential determination

DiOC_2_(3) was used to demonstrate the alteration of bacterial membrane potential. When encountering intracellular less-negative electrical membrane potential, such fluorochrome tended to self-polymerase accompanied by its emission wavelength shifting from green to red fluorescence. To test the membrane potential, bacteria growing to the mid-log phase were harvested and processed in EDTA (10 mM) for 5 min to allow the dye to penetrate the inner membrane (Hudson et al., 2020). The bacteria were then spun to collect the pellets and resuspended in sTSB mimicking the same culture condition. After incubation with DiOC_2_(3) (30 μM) for 30 min, the red/green fluorescence ratios of *P. gingivalis* WT, Δ*trkA*, and Δ*trkA*/*trkA* were normalized to those of WT treated with 0.5% DMSO and 5 μM of CCCP, each alone. The formula used is as follows:


$${\text{Fluor}}_{\text{normalized}}=\left({\text{Fluor}}_{\text{sample}}-{\text{Fluor}}_{\text{CCCP}}\right)/\left({\text{Fluor}}_{\text{CCCP}}-{\text{Fluor}}_{\text{DMSO}}\right)$$

Fluor_sample_ represents the red/green fluorescence ratio of the samples, while Fluor_CCCP_ and Fluor_DMSO_ represent the positive and negative controls (Zhang et al., 2020).

### Identification of heme-bound ability to *Porphyromonas gingivalis* cells

*Porphyromonas gingivalis* WT, Δ*trkA*, and Δ*trkA*/*trkA* were grown in the sTSB medium to OD_600_ of 0.8–1.2. Cultures were then centrifuged (9,000*g* for 10 min), washed, and resuspended in PBS to the adjusted OD_600_ of 1.0. The bacterial suspension (800 μl) was then mixed with 200 μl of 50 μM heme. After incubation at 37°C for 1 h, the samples were centrifuged and decreased optical density was determined at 380 nm (Liu et al., 2006). The heme diluted in 800 μl of PBS was used as the negative control.

### qRT-PCR

Total RNA was extracted from *P. gingivalis* WT, Δ*trkA*, and Δ*trkA*/*trkA* using RNAiso Plus (TaKaRa, Kusatsu, Japan) and transcribed into cDNA by PrimeScript™ RT reagent kit with gDNA Eraser (TaKaRa, Kusatsu, Japan). qRT-PCR was performed on the QuantStudio 6 Flex (Applied Biosystems,Carlsbad , USA) using TB Green^®^ Premix Ex Taq™ II (TaKaRa, Kusatsu, Japan). The 16S rRNA gene was used as the endogenous control, and relative expression counts were calculated. All primers used are listed in **Table S1**.

### Virulence assay *in vivo*

Virulence *in vivo* of *P. gingivalis* WT and Δ*trkA* was compared in terms of murine alveolar bone loss. Briefly, 6-week-old female mice were obtained from the Ensiweier Laboratory and fed with a standard diet. All experiments involving animals were approved by the Ethics Committee of West China Hospital of Stomatology. *Porphyromonas gingivalis* WT and Δ*trkA* strains of 10^10^ CFUs were resuspended in 100 μl of PBS buffer containing 2% carboxymethylcellulose (CMC) and orally inoculated into randomly assigned mice with a 2-day interval for 12 days (Shen et al., 2020). As for the blank control group, an equivalent volume of PBS only with 2% CMC was used. Forty-two days after the last bacteria inoculation, mice were euthanized and maxillary bones were then scanned by microcomputed tomography (Hiscan XM). Bone volume fraction, trabecular thickness, trabecular number, and trabecular separation were measured with the help of the software Hiscan Analyzer. Bone resorption was analyzed by measuring the distance from the cementoenamel junction to the alveolar bone crest at 14 sites of three molars.

### Statistical analysis

Statistical analysis was conducted using GraphPad Prism software version 7.0. Unpaired two-tailed *t*-tests were performed to analyze the differences between the two groups. For data from two groups with no homogeneity of variance, Welch’s *t*-test was carried out instead. Ordinary one-way analysis of variance (ANOVA) was used to measure the differences of more than two groups, while Tukey’s test was added for further multiple comparisons. Data are presented as mean ± standard deviation (mean ± SD). Statistical significance was set at *p*< 0.05.

## Results

### Role of the *trkA* gene in the transcriptomic level

**Figure 1:**
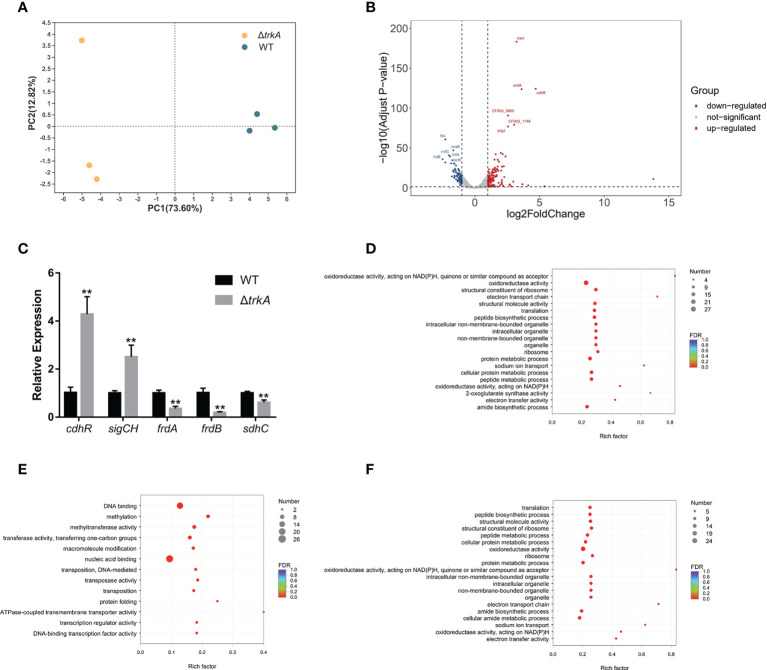
Transcriptional profiling of *Porphyromonas gingivalis* Δ*trkA* versus the wild-type (WT) strain. **(A)** Principal component analysis of *P. gingivalis* Δ*trkA* versus the WT strain shows a clear divergence. **(B)** Volcano plot of differentially expressed genes. **(C)** qRT-PCR analysis of a subset of differentially expressed genes detected by RNA sequencing. Gene ontology enrichment analysis of **(D)** differentially expressed genes, **(E)** upregulated genes, and **(F)** downregulated genes in the *P. gingivalis* Δ*trkA* strain. Data are presented as means ± SD, normalized to 16S rRNA and compared to WT (2^−ΔΔCT^). *N* = 3, **p< 0.01.

To comprehensively investigate how the *trkA* gene functioned in *P. gingivalis*, we compared the transcriptomic difference between wild-type and Δ*trkA* strains by applying bulk RNA sequencing. Principal component analysis showed apparent separation between the wild-type and Δ*trkA* strain groups (**Figure 1A**). To figure out which genes contributed to such difference, we curated a list of differentially expressed genes with a threshold of log_2_ fold change ≥1 and adjusted *p*-value<0.05 (**Tables S2**, **S3**). As shown in **Figure 1B**, the top 5 differentially expressed genes with the lowest adjusted *p*-value were noted among 129 upregulated and 86 downregulated genes. Differential expression of some genes and subsets was verified by qRT-PCR (**Figure 1C**).

We then mapped differentially expressed genes to the GO database for enrichment analysis (**Figure 1D**), since almost 72% of those genes were annotated in the database. It was accordingly found that downregulated genes were mainly enriched during translation, sodium ion transport, oxidoreductase activity, and electron transport chain (ETC) (**Figure 1F**). The regulated genes mapped to sodium ion transport were *nqrABCDEF*, considered as the putative complex I of ETC (Meuric et al., 2010). Additionally, another candidate gene encoding complex I, *rnfABCDGE*, was also downregulated together with the complex II regulon, *frdAB*, and *sdhC*. The latter protein complex was reckoned as the final electron receptor during anaerobic respiration (Spinelli et al., 2021), converting fumarate to succinate for further fermentation, hence associated with putative alteration of metabolic profile. For upregulated genes, there was an overrepresentation of GO terms related to DNA binding, methylation, and transferase activity of transferring one-carbon groups (**Figure 1E**). Of note, *cdhR* was the most upregulated gene among a series of DNA-binding and transcriptional factors. Such gene encoding protein acted as an upstream regulator of heme uptake regulon *hmuYR* (Wu et al., 2009), indicating a putative modification of the heme acquisition process after deleting *trkA*.

### *trkA* knockout strain exhibits reduced hemagglutination ability

**Figure 2:**
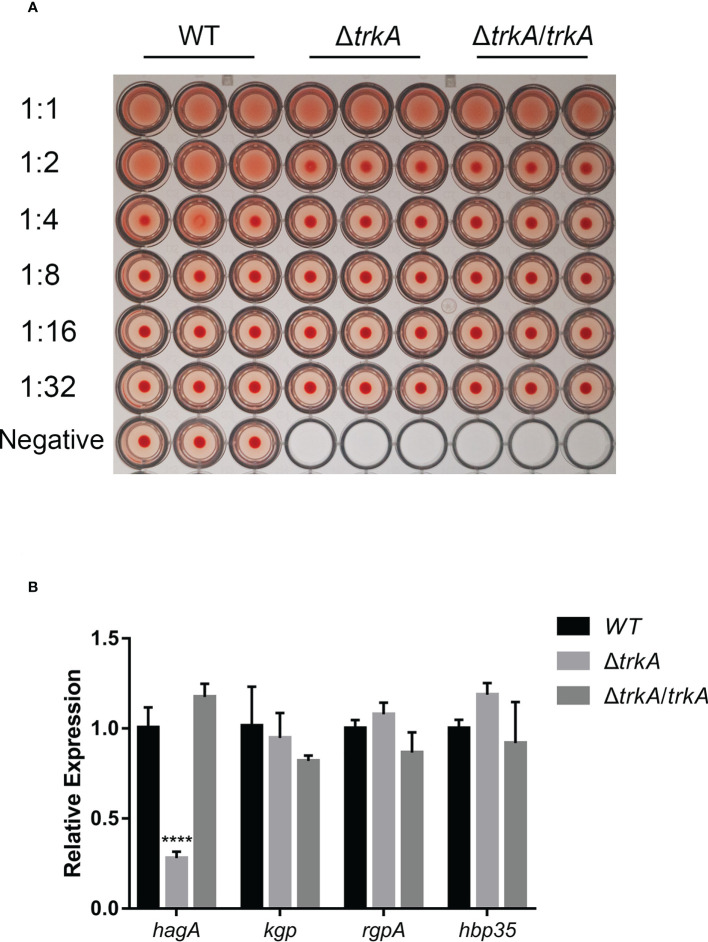
*trkA* deletion attenuates hemagglutination activity of *Porphyromonas gingivalis*. **(A)** Hemagglutination activity of *P. gingivalis* WT, Δ*trkA*, and Δ*trkA*/*trkA* strains. **(B)** qRT-PCR analysis of *hagA*, *kgp*, *rgpA*, and *hbp35* mRNA expression in *P. gingivalis* WT, Δ*trkA*, and Δ*trkA*/*trkA* strains. Data are presented as means ± SD, normalized to 16S rRNA and compared to WT (2^−ΔΔCT^). *N* = 3, ****p< 0.001.

Since hemagglutination plays a critical role in heme acquisition and virulence, we began by measuring the hemagglutinate ability of *P. gingivalis* after deleting the gene *trkA*. As shown in **Figure 2A**, the Δ*trkA* strain displayed hemagglutination titers one to two dilutions lower than the wild-type strain. We further compared the transcript level of protein containing the heme-binding domain (HA2). qRT-PCR analysis showed that the mRNA level of the *hagA* gene encoding hemagglutinin was decreased, while complementation of *trkA* rescued *hagA* expression. The expression of other genes encoding proteins with adhesion domains such as *rgpA*, *kgp*, and *hbp35* was unaffected (**Figure 2B**). Thus, downregulated *hagA* might be the determinant of reduced hemagglutination activity in Δ*trkA* strains, indicating the correlated function between the *hagA* and *trkA* genes.

### The *trkA* knockout strain displays membrane potential-related hemolysis activity alteration

**Figure 3:**
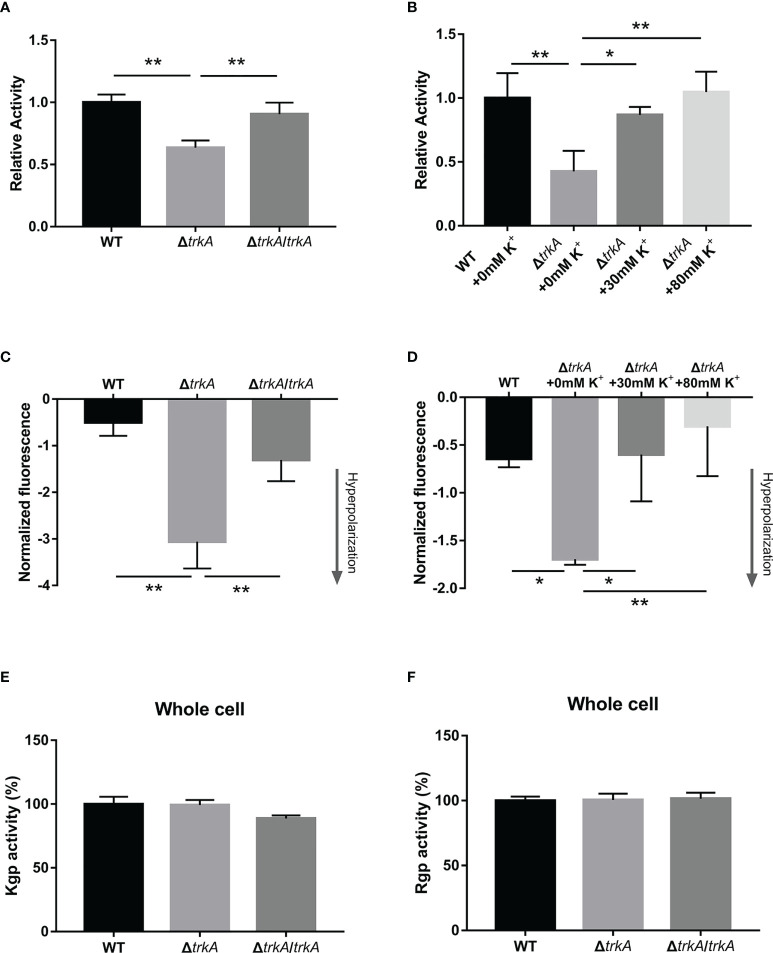
Loss of *trkA* leads to membrane potential-related hemolysis activity alteration. **(A, B)** Hemolytic assay, **(C, D)** membrane potential determination, and **(E, F)** Kgp and Rgp activities of *Porphyromonas gingivalis* WT, Δ*trkA*, and Δ*trkA*/*trkA* strains. *N* = 3, *p< 0.05, **p< 0.01.

*Porphyromonas gingivalis* releases free heme sources from hemoglobulin-contained erythrocytes much more efficiently than from albumin and hemopexin when residing in the inflammatory pocket (Shizukuishi et al., 1995), such that hemolysis works as the main pathway during the heme acquisition process. To test whether there was an alteration of hemolysis after deleting *trkA*, we compared the hemolytic activity of the wild-type, Δ*trkA*, and Δ*trkA*/*trkA* strains by calculating the released amount of hemoglobulin into the supernatant during incubation with erythrocytes. As shown in **Figure 3A**, the relative activity of the Δ*trkA* strain group showed an apparent downward tendency to approximately 64% of that in the wild-type and Δ*trkA*/*trkA* strain groups. Gingipain activity was later assessed, as it accounts for 85% of the extracellular proteolytic activity of *P. gingivalis* and is responsible for hemolysis (Potempa et al., 2003; Li et al., 2010). However, there was no difference in arginine- and lysine-specific gingipain activity among *P. gingivalis* WT, Δ*trkA*, and Δ*trkA*/*trkA* strains (**Figures 3E, F**).

Notably, the relative hemolytic activity of the Δ*trkA* strain could be augmented when adding a certain concentration of potassium ion, the extent to which there was no statistically significant difference between the Δ*trkA* strain in the medium (80 mM of potassium ion) and the wild type cultured without extra potassium added (*p* = 0.9935) (**Figure 3B**). Thus, there is a certain relationship between hemolytic activity and *trkA* as well as extracellular potassium. Considering that the *trkA* gene, encoding the potassium transport regulatory protein, can control the influx and efflux of potassium across the membrane (Cao et al., 2013), we subsequently measured the status of membrane potential in response to *trkA* deleted and variant extracellular potassium ion. It was found that the *P. gingivalis* Δ*trkA* strain exhibited more hyperpolarized membrane potential (**Figure 3C**), in line with our hypothesis that less potassium influx was permitted in the case of *trkA* loss and, thus, more negative membrane potential could be displayed. Similar to the alteration of extracellular potassium-related hemolytic activity, the extent of hyperpolarization was gradually attenuated as the extracellular potassium concentration increased (**Figure 3D**). Accordingly, the hyperpolarization of membrane potential could partially explain the diminished hemolytic activity.

### Loss of *trkA* exerts no effect on heme-bound capacity but is related to gene transcripts

**Figure 4:**
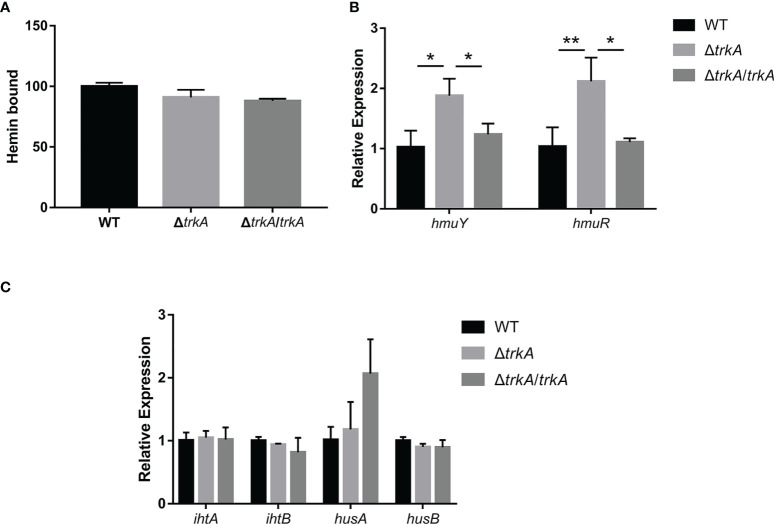
Heme-bound capacity of *Porphyromonas gingivalis* is not altered except for the corresponding transcripts. **(A)** Heme-bound assay. qRT-PCR of **(B)** *hmuYR* and **(C)** *ihtAB* and *husAB* mRNA expression of *P. gingivalis* WT, Δ*trkA*, and Δ*trkA*/*trkA* strains. Data are presented as means ± SD, normalized to 16S rRNA and compared to WT (2^−ΔΔCT^). *N* = 3, *p< 0.05, **p< 0.01.

Heme liberated from hemoglobulin would adhere to the bacterial surface and be transferred across the outer membrane into the cells. Therefore, we ulteriorly estimated the effect of *trkA* on the heme-bound capacity in *P. gingivalis*. The heme-bound assay depicted that there was no apparent difference among the wild-type, Δ*trkA*, and Δ*trkA*/*trkA* strains (**Figure 4A**). Such phenotype result was not in congruency with related transcriptomic alteration. As is uncovered of the highly upregulated expression of the *cdhR* gene, encoding the transcriptional regulator of *hmuYR*, in transcriptomic analysis, we further tested the implication on *hmuYR* transcripts after deleting *trkA*. In accordance with upregulated *cdhR*, the mRNA level of *hmuYR* was increased (**Figure 4B**). Additionally, the expression of other genes encoding the heme transporter complex was unaffected, and there was exclusion of compensation effect (**Figure 4C**). Thus, the paradox between transcripts and phenotype awaits further investigation.

### *In vivo* pathogenicity determination of *Porphyromonas gingivalis* Δ*trkA* strain

**Figure 5:**
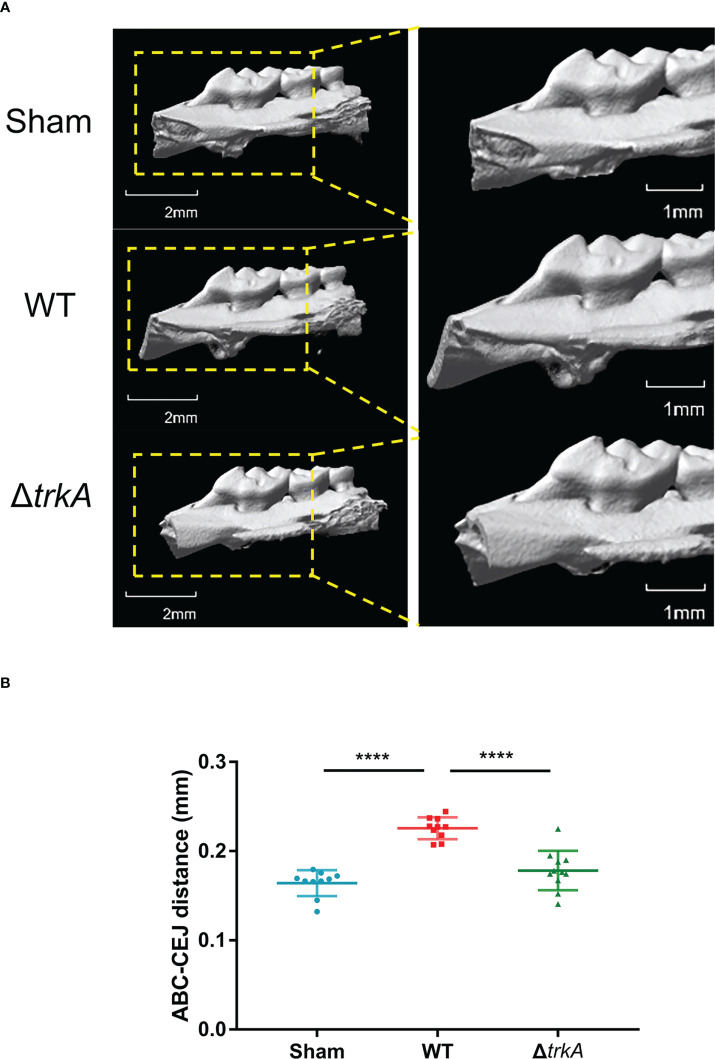
*trkA* is required for the virulence of *Porphyromonas gingivalis in vivo*. Micro-CT analysis of alveolar bone loss in mice following infection with *P. gingivalis* wild-type and Δ*trkA* strains. The group treated with CMC (sham) worked as a control. **(A)** Three-dimensional micro-CT reconstruction of the alveolar bone and **(B)** analysis of bone resorption. All data of the distance between ABC and CEJ in 14 sites of the first, second, and third molars were measured and presented as mean ± SD. ABC, alveolar bone crest; CEJ, cementoenamel junction. *N* = 10 or 11, ****p< 0.001.

The pathogenicity of the *P. gingivalis* Δ*trkA* strain was evaluated by measuring murine alveolar bone resorption after oral inoculation for a period. As shown in the three-dimensional reconstruction of bone through micro-CT (**Figure 5**), mice inoculated with the *P. gingivalis* wild-type strain exhibited reduced bone loss level compared with the control group treated with 2% CMC. Deletion of *trkA* displayed alleviated bone resorption. Additionally, the mutant strain showed abated levels of bone volume fraction (BV/TV), trabecular number (Tb.N), and trabecular separation (Tb.Sp) compared with the wild-type strain group (**Figure S1**). Thus, *trkA* can control the optimal virulence of *P. gingivalis* to mediate oral infection.

## Discussion

Potassium accounts for the most abundant inorganic cation in the cytoplasm, and its uptake is crucial for multiple basic cellular activities (Epstein, 2003; Beagle and Lockless, 2021; Do and Gries, 2021). Apart from the physiological functions of osmotic and pH homeostasis, the putative pathological roles of potassium ion uptake systems have been successively well-reported in various bacteria species, whereas few are hitherto investigated in periodontal-related pathogens, such as *P. gingivalis*. According to retrieval from the KEGG database, *trkA* and *trkH* are genes involved in potassium transport in *P. gingivalis*. TrkA of *Vibrio parahaemolyticus* has been reported to change its conformation from tetramer to dimer when binding to nucleotides such as ATP in the regulation of the opening frequency of the TrkH ion channel and, thus, works as a regulator of cation flux and then impacts other biological processes (Zhang et al., 2020). The TrkA protein from *P. gingivalis* shares 47% similar matches to *V. parahaemolyticus*, while the ARG98 residue responsible for binding to γ-phosphate of ATP (Cao et al., 2013) is conserved in both species based on the NCBI Protein BLAST. It is then plausible that the TrkA protein might act in a similar way as observed in *V. parahaemolyticus*. In this study, we initially reported a correlation between the gene-encoding potassium transport regulatory protein TrkA and the heme acquisition pathway, especially in the hemagglutination and hemolysis process of *P. gingivalis*, demonstrating the role of the *trkA* gene in maintaining optimal virulence during murine oral infection.

The heme acquisition process is an indispensable way for *P. gingivalis* to multiply and exhibit pathogenicity. It has been demonstrated that *P. gingivalis* displayed a changeable growth rate in the influence of heme concentration in the culture medium (Marsh et al., 1994) and enhanced virulence *in vivo* in a heme dose-dependent manner (McKee et al., 1986). Elementally, heme accumulates onto the cell surface of *P. gingivalis* in a μ-oxo bisheme form to catalyze excess H_2_O_2_ released by activated neutrophils (Smalley et al., 2000). Meanwhile, ferrous ions liberated from heme can be utilized and integrated into an iron–sulfur cluster involving ETC and energy metabolism (Olczak et al., 2005). Moreover, increased heme added into the culture would also bring about energy metabolism conversion in relation to more metabolites such as succinate, propionate, and butyrate, thus augmenting the virulence *in vivo* (McKee et al., 1986).

*Porphyromonas gingivalis* mainly takes erythrocytes as the source for heme utilization. Physiologically, heme from free hemoglobulin as a result of cell lysis is rapidly liberated and scavenged by albumin and hemopexin which display higher affinity. Therefore, *P. gingivalis* would proactively undergo the procedure in the acquisition of heme through adherence to erythrocytes, hemolysis, heme uptake, and storage (Smalley and Olczak, 2017). In the isogenic Δ*trkA* strain, we observed alleviated hemagglutinate and hemolytic activity along with downregulated *hagA* expression and membrane potential alteration, which indicated that the basic steps of the heme acquisition process were impaired. Interestingly, we detected a distinct downregulation of the *hagA* gene, which is considered as the main attribute to adherence to erythrocytes (Han et al., 1996) and to stabilize the porUV sortase structure of type IX secretion system for the normal secretion of proteins like gingipains (Saiki and Konishi, 2014). The mechanism of *hagA* regulation by *trkA* is unclear. It was reported that the ferric uptake regulation protein homolog (PgFur) positively controlled *hagA* expression (Ciuraszkiewicz et al., 2014). Another mechanism involving the regulation of *hagA* expression is the two-component system haeSR regulon which was determined to bind to the upstream sequence of the *hagA* gene according to the ChIP-Seq experiment (Scott et al., 2013). *haeSR* and *PgFur* were both unaffected pursuant to our RNA-seq data, which indicated the downstream role of TrkA in the HaeSR– and PgFur–hagA regulatory axis, or there might be another candidate independent signaling. So far, further study is needed to elucidate the relation between the *trkA* and *hagA* genes.

In the control of cation flux, TrkA and its associated protein TrkH can impact the membrane potential. In line with multiple studies (Gries et al., 2013; Cholo et al., 2015), our result showed a more hyperpolarized membrane potential and potassium-related repolarization in the isogenic Δ*trkA* strain, supporting the hypothesis of the important role of TrkA in regulating potassium flux into the cytoplasm and causing a less hostile and negative intracellular environment. Earlier experiments have elucidated that various factors would contribute to the alteration of hemolytic activity, including magnesium, calcium, protease inhibitors, and metabolic inhibitors. Notably, one of them, CCCP, also brings about the depolarization of the membrane potential (Chu et al., 1991). Thus, a certain membrane potential is crucial for *P. gingivalis* to maintain optimal hemolytic activity. It is of much concern that membrane potential could give rise to multiple impacts on bacteria, including cell growth (Stratford et al., 2019), antibiotic resistance (Gries et al., 2013), and biofilm formation (Prindle et al., 2015). Those alterations were also recognized in the isogenic Δ*trkA* strain (data not shown) with retarded growth and tiny single colony formation, which needs further analysis.

The diminished heme acquisition process would possibly induce lower virulence *in vivo*, in accordance with what we detected in the murine model of experimental periodontitis. To have a comprehensive understanding of function disorder and supplement extra information on the mechanisms underlying virulence changing caused by *trkA* deletion in *P. gingivalis*, we performed transcriptomic analysis between the wild-type and isogenic Δ*trkA* strains. Following enrichment analysis and retrieval for individual genes, genes encoding ETC were mostly downregulated. As the pathway involving energy metabolism, ETC has been deduced of giving way to substrate level phosphorylation during anaerobic respiration in *P. gingivalis* by providing succinate and facilitating the formation of the high-energy compound butyryl-CoA through the ferredoxin and butyryl-CoA dehydrogenase/electron transfer flavoprotein (Bcd/EtfAB) complex (Meuric et al., 2010). As such, we hypothesized that the downregulation of genes encoding complexes I and II would disrupt the output of metabolites like butyrate and acetate, which might partly affect the holistic virulence of *P. gingivalis*. Of note, *cdhR* was the most upregulated gene and linked to the heme uptake process. As a transcriptional regulator, it is believed to control *hmuYR* expression (Wu et al., 2009), in agreement with the transcript result in our study. However, the phenotype comparison was not congruent with the mRNA level. We inferred that such paradox would be ascribed to a weak protein biosynthesis. Deletion of the potassium transporter can give rise to a decreased level of intracellular potassium ion (Durand et al., 2016), which has been elucidated to be involved in ribosome assembling and functions. Structurally, potassium has been reported to maintain mRNA within the decoding center during the elongation state of translation and to stabilize tRNA, rRNA, and subunits of the ribosome (Rozov et al., 2019), and loss of potassium can lead to absent poly-Phe polymerization activity *in vitro* (Naslund and Hultin, 1970). Furthermore, the potential alteration of posttranslational modification and subcellular localization remains obscure. Accordingly, more investigations such as on the protein concentration of whole cells, membrane part, and cytosol need to be further determined. In that regard, the upregulated expression of *cdhR* might be a negative feedback to impaired heme uptake process in the case of insufficient potassium influx of the Δ*trkA* strain. Additionally, *cdhR* was also reported to control *P. gingivalis* microcolony formation on the substratum of *Streptococcus gordonii*. The initial attachment to *S. gordonii* will lead to a sequence of protein tyrosine (de)phosphorylation which gives rise to phosphorylation of CdhR along with increased nososymbiocity (Lamont et al., 2018). Although we observed a high level of *cdhR* mRNA, the protein level and posttranscriptional modification were not determined heretofore.

Collectively, we focus for the first time on the *trkA* effect on *P. gingivalis* W83, as it belongs to the virulent strains of *P. gingivalis* according to the murine subcutaneous soft tissue abscess model analysis (Slots and Rams, 1993). The potential function variance of *trkA* in other strains of *P. gingivalis* such as ATCC33277, HG66, and TDC60 needs further study. Moreover, it remains elusive what attributes in the *P. gingivalis* Δ*trkA* strain account for the reduced pathogenicity. More investigations need to be made such as comparison in terms of single or multiple species biofilm formation and *in-vitro* analysis of metabolic profile as well as under various stresses like intracellular survival to understand the regulatory mechanism of TrkA.

## Data availability statement

The datasets presented in this study can be found in online repositories. The names of the repository/repositories and accession number(s) can be found below: NCBI, GSE210663.

## Ethics statement

The animal study was reviewed and approved by the Ethics Committee of West China Hospital of Stomatology, Sichuan University.

## Author contributions

RZ contributed to the study conceptualization, data acquisition, analysis, interpretation, and writing of the original draft. LZ contributed to the study design and interpretation. DS and YW contributed to the study conceptualization, design, interpretation, and critical revision of the manuscript. All authors approved the final version of the manuscript and accounted for all aspects of the work.

## Funding

This study was supported by the National Natural Science Foundation of China (grant number 82170970).

## Conflict of interest

The authors declare that the research was conducted in the absence of any commercial or financial relationships that could be construed as a potential conflict of interest.

## Publisher’s note

All claims expressed in this article are solely those of the authors and do not necessarily represent those of their affiliated organizations, or those of the publisher, the editors and the reviewers. Any product that may be evaluated in this article, or claim that may be made by its manufacturer, is not guaranteed or endorsed by the publisher.
