# Supplementary material for: TrkA serves as a virulence modulator in Porphyromonas gingivalis by maintaining heme acquisition and pathogenesis
Source: Front Cell Infect Microbiol. 2022 Nov 2;12:1012316. doi: 10.3389/fcimb.2022.1012316 (PMC9666725; doi:10.3389/fcimb.2022.1012316)
Supplement: Supplementary file 1 [file DataSheet_1.docx]

Supplementary Material

Table S1 Primers used in this study

| Primer name |  | Primer sequence (5’ – 3’) | Function |
| --- | --- | --- | --- |
| trkA_Up794 | F | AAGGGTTGATTCCGTTTTGC | Generation of Δ*trkA* |
|  | R | AGCTATCGCTTGCTACATTAAGGTTTGTAAGATC |  |
| trkA_Down800 | F | CGATGGAGCCCTCTCTTTTCGGACGGC |  |
|  | R | GATGCTGAGACTGCCGTA |  |
| trkA_promoter_BamHⅠ | F | GCATCGGGATCCGAGAGAGGTCGATAGGAAAA | Cloning of *trkA* and into pT-COW |
| trkA_ORF_salI | R | CGTAGCGTCGACTTAGCCGAAGAAGTCTTTCAGG |  |
| cdhR | F | CCACGCCACAGTAGAGGAAT | qPCR |
|  | R | TCTCTTTCTCCCGATTGGTG |  |
| sigCH | F | TACTGGAGCAATCCGAGACC | qPCR |
|  | R | GCTTCGACTTTCACGCCTAC |  |
| frdA | F | TTGCCGTATTCCTCGACTTC | qPCR |
|  | R | CGGATTCTCATCTGCGATCT |  |
| frdB | F | AGAGACCTCATGGTGGATCG | qPCR |
|  | R | CGCATTCTTCTTGGAAATGG |  |
| sdhC | F | TCCGCTACACATTCAACCAA | qPCR |
|  | R | TTGTTCCATCCCAAGGTCTG |  |
| hagA | F | GGGTGTCTCCAAAGGTAACGA | qPCR |
|  | R | CACTACAACGGACGCAGCA |  |
| kgp | F | AGGAACGACAAACGCCTCTAT | qPCR |
|  | R | CACCAACCAAAGCCAAGAAG |  |
| rgpA | F | GACAAGGACCGACGAAAGAA | qPCR |
|  | R | TAATCGCTTCCACCACCTTC |  |
| hbp35 | F | GGATCAAACATTGGGCATTC | qPCR |
|  | R | GGGCTGTACCATCACCAAAA |  |
| hmuY | F | TACGAAATGGGACCTGATGG | qPCR |
|  | R | CCAACCACCTGAAGCAAATC |  |
| hmuR | F | GCGACGGACAGAAATACGAT | qPCR |
|  | R | GCCTGCAACATTCAGTTCCT |  |
| ihtA | F | ATCTCCAGACGGATGAGTGG | qPCR |
|  | R | AAAGGCTCACGCCAAGTCTA |  |
| ihtB | F | CGAGTCCGATCCCTCTATCA | qPCR |
|  | R | ACGTCTTCCGACTATCGTCCT |  |
| husA | F | AGCCTGTTTGCGTAGCATG | qPCR |
|  | R | GATTAGCAGGGTTGGCTTCA |  |
| husB | F | CAGAACAAGACGGTGCTCAA | qPCR |
|  | R | CTCACGATCTCCCACATCCT |  |

Table S2 Upregulated genes in *P. gingivalis* W83 Δ*trkA* strain

| Gene ID | Gene name | Log2FC | P value | Regulate |
| --- | --- | --- | --- | --- |
| CF003_2219 | trkH | 3.222420502 | 4.52E-184 | Up |
| CF003_1237 | cdhR | 4.704806767 | 6.19E-125 | Up |
| CF003_1236 | scdA | 3.612042835 | 1.89E-124 | Up |
| CF003_0865 | CF003_0865 | 2.566188145 | 2.61E-91 | Up |
| CF003_1746 | CF003_1746 | 3.038825394 | 8.52E-80 | Up |
| CF003_1870 | ycgJ | 2.569545952 | 1.46E-77 | Up |
| CF003_1350 | CF003_1350 | 2.005236303 | 1.62E-44 | Up |
| CF003_1181 | betI_1 | 2.152584602 | 4.84E-43 | Up |
| CF003_2101 | CF003_2101 | 1.613505102 | 1.12E-42 | Up |
| CF003_0778 | tsaB | 2.373950043 | 5.28E-40 | Up |
| CF003_1335 | CF003_1335 | 1.523431581 | 4.65E-36 | Up |
| CF003_1682 | yqgM | 1.766611143 | 7.01E-36 | Up |
| CF003_1240 | kstR2 | 1.853912935 | 1.65E-33 | Up |
| CF003_1175 | ndvA_1 | 1.855186871 | 2.37E-33 | Up |
| CF003_1829 | fadD15 | 2.236583914 | 1.31E-27 | Up |
| CF003_1180 | ydfJ_1 | 1.768153233 | 2.21E-27 | Up |
| CF003_1178 | CF003_1178 | 2.097569696 | 3.45E-27 | Up |
| CF003_1019 | CF003_1019 | 1.552588575 | 8.00E-26 | Up |
| CF003_1055 | tpr_7 | 1.660578208 | 3.57E-24 | Up |
| CF003_1176 | irtA_1 | 1.489987444 | 4.43E-24 | Up |
| CF003_1681 | glgX | 1.103962959 | 7.04E-24 | Up |
| CF003_1000 | alkD | 1.741048622 | 7.04E-24 | Up |
| CF003_2061 | dfrA | 1.379963438 | 9.62E-24 | Up |
| CF003_0856 | CF003_0856 | 1.737661605 | 1.02E-23 | Up |
| CF003_2102 | tapA | 1.484417344 | 2.12E-23 | Up |
| CF003_n10 | CF003_n10 | 2.032664306 | 2.35E-22 | Up |
| CF003_1602 | CF003_1602 | 1.595423108 | 3.64E-22 | Up |
| CF003_0798 | CF003_0798 | 1.810638756 | 5.93E-22 | Up |
| CF003_0418 | clpP | 1.35785192 | 2.83E-21 | Up |
| CF003_0573 | rsmH | 1.060008412 | 5.05E-21 | Up |
| CF003_1179 | CF003_1179 | 1.642534973 | 5.18E-21 | Up |
| CF003_0029 | spoU | 1.510616444 | 3.66E-20 | Up |
| CF003_n28 | CF003_n28 | 1.373550954 | 3.80E-20 | Up |
| CF003_1326 | kgp_2 | 1.289261786 | 6.42E-20 | Up |
| CF003_1211 | dapH | 1.395310032 | 3.07E-19 | Up |
| CF003_1764 | fabF | 1.110221549 | 7.16E-19 | Up |
| CF003_1212 | bepA_2 | 1.281467927 | 2.69E-17 | Up |
| CF003_1189 | CF003_1189 | 1.003697356 | 9.49E-17 | Up |
| CF003_1001 | CF003_1001 | 1.692560377 | 1.33E-16 | Up |
| CF003_0997 | hipB_1 | 1.437474331 | 2.78E-16 | Up |
| CF003_1775 | grpE | 1.318153238 | 4.26E-16 | Up |
| CF003_1927 | rplX | 1.145327585 | 4.98E-16 | Up |
| CF003_1469 | hsdM | 1.153151932 | 6.67E-16 | Up |
| CF003_1308 | CF003_1308 | 1.318103695 | 1.43E-15 | Up |
| CF003_1032 | CF003_1032 | 1.697527711 | 2.21E-15 | Up |
| CF003_0963 | CF003_0963 | 1.519504621 | 3.18E-15 | Up |
| CF003_0849 | CF003_0849 | 1.545655668 | 3.81E-15 | Up |
| CF003_n25 | CF003_n25 | 1.599022443 | 4.29E-15 | Up |
| CF003_1765 | acpP | 1.0693935 | 6.98E-15 | Up |
| CF003_0860 | CF003_0860 | 1.58238124 | 1.53E-14 | Up |
| CF003_1880 | mftF | 1.042018479 | 2.52E-14 | Up |
| CF003_1465 | CF003_1465 | 2.27669102 | 3.60E-14 | Up |
| CF003_2048 | CF003_2048 | 1.172506727 | 4.33E-14 | Up |
| CF003_1696 | llaBIIM | 1.148406864 | 5.47E-14 | Up |
| CF003_0389 | nusG | 1.065929296 | 6.90E-14 | Up |
| CF003_1745 | thrS_1 | 1.076689806 | 1.17E-13 | Up |
| CF003_0999 | CF003_0999 | 1.417605316 | 2.77E-13 | Up |
| CF003_1213 | rnhA | 1.429469099 | 3.12E-13 | Up |
| CF003_2030 | CF003_2030 | 1.09103007 | 4.75E-13 | Up |
| CF003_0968 | mrr | 1.315957978 | 1.03E-12 | Up |
| CF003_1857 | CF003_1857 | 1.191123084 | 1.08E-12 | Up |
| CF003_1879 | yqhO | 1.010948752 | 1.11E-12 | Up |
| CF003_2169 | CF003_2169 | 1.093057482 | 1.48E-12 | Up |
| CF003_0574 | ftsL | 1.085950867 | 3.18E-12 | Up |
| CF003_1020 | tonB_4 | 1.174185875 | 4.77E-12 | Up |
| CF003_n40 | CF003_n40 | 1.175817363 | 6.34E-12 | Up |
| CF003_2176 | CF003_2176 | 13.80241236 | 1.17E-11 | Up |
| CF003_0565 | asxL | 1.392920981 | 1.53E-11 | Up |
| CF003_1142 | epsG | 1.032930158 | 2.68E-11 | Up |
| CF003_0918 | CF003_0918 | 1.352565239 | 7.92E-11 | Up |
| CF003_1493 | cnaB_3 | 1.078453984 | 1.04E-10 | Up |
| CF003_0847 | CF003_0847 | 2.101433902 | 1.46E-10 | Up |
| CF003_0346 | engB | 1.275005505 | 1.80E-10 | Up |
| CF003_0719 | haeS | 1.247595736 | 1.96E-10 | Up |
| CF003_0423 | CF003_0423 | 1.06829307 | 2.07E-10 | Up |
| CF003_0271 | ssb | 1.107873651 | 2.50E-10 | Up |
| CF003_0521 | groS | 1.13418393 | 3.06E-10 | Up |
| CF003_1499 | CF003_1499 | 1.045749511 | 3.13E-10 | Up |
| CF003_0261 | CF003_0261 | 1.46840367 | 3.66E-10 | Up |
| CF003_1683 | amyA_1 | 1.179871016 | 5.37E-10 | Up |
| CF003_1026 | CF003_1026 | 1.387286208 | 8.59E-10 | Up |
| CF003_n26 | pssA | 1.372857569 | 1.15E-09 | Up |
| CF003_0181 | mfa4 | 1.204015463 | 2.34E-09 | Up |
| CF003_1118 | clpB | 1.225147954 | 2.77E-09 | Up |
| CF003_1262 | CF003_1262 | 1.093065593 | 3.30E-09 | Up |
| CF003_0834 | CF003_0834 | 1.072662602 | 4.56E-09 | Up |
| CF003_2092 | CF003_2092 | 1.012555915 | 5.32E-09 | Up |
| CF003_1238 | truC | 1.394739102 | 6.45E-09 | Up |
| CF003_0866 | CF003_0866 | 1.080749109 | 7.11E-09 | Up |
| CF003_0480 | cobI | 1.06575466 | 7.95E-09 | Up |
| CF003_1512 | CF003_1512 | 2.976201681 | 2.00E-08 | Up |
| CF003_1601 | birA | 1.036149585 | 4.34E-08 | Up |
| CF003_1466 | icmt | 2.060297277 | 7.12E-08 | Up |
| CF003_0930 | vgrG | 1.212926285 | 8.59E-08 | Up |
| CF003_1003 | acg | 1.129530125 | 9.20E-08 | Up |
| CF003_1447 | btr_1 | 2.087974649 | 1.22E-07 | Up |
| CF003_0826 | btr_2 | 2.087974649 | 1.22E-07 | Up |
| CF003_0214 | sigCH | 1.166149512 | 1.28E-07 | Up |
| CF003_0682 | macB_3 | 1.073090406 | 3.87E-07 | Up |
| CF003_0848 | CF003_0848 | 1.246557665 | 5.71E-07 | Up |
| CF003_1467 | ubiE_2 | 1.827467763 | 6.46E-07 | Up |
| CF003_1318 | sigW_3 | 1.013842058 | 1.59E-06 | Up |
| CF003_0680 | bepF | 1.070151596 | 8.47E-06 | Up |
| CF003_1503 | lytB | 2.08929736 | 1.48E-05 | Up |
| CF003_0871 | CF003_0871 | 2.06105192 | 2.30E-05 | Up |
| CF003_1534 | CF003_1534 | 3.672084103 | 3.63E-05 | Up |
| CF003_1505 | rSAM | 2.673541266 | 7.24E-05 | Up |
| CF003_1617 | CF003_1617 | 2.718767716 | 0.000215599 | Up |
| CF003_0846 | CF003_0846 | 1.083289228 | 0.000276896 | Up |
| CF003_1489 | mobB | 1.492572964 | 0.000299273 | Up |
| CF003_1165 | CF003_1165 | 1.374512026 | 0.000383411 | Up |
| CF003_1359 | CF003_1359 | 1.032160669 | 0.000424808 | Up |
| CF003_1929 | rpsQ | 1.30889447 | 0.000557266 | Up |
| CF003_0917 | gtrA | 1.231181273 | 0.000657501 | Up |
| CF003_1461 | CF003_1461 | 4.130896011 | 0.000858625 | Up |
| CF003_0870 | CF003_0870 | 1.9403381 | 0.00087059 | Up |
| CF003_0216 | CF003_0216 | 1.191587938 | 0.000996558 | Up |
| CF003_1470 | CF003_1470 | 1.379089879 | 0.002459673 | Up |
| CF003_0875 | CF003_0875 | 2.183582795 | 0.00261471 | Up |
| CF003_2194 | CF003_2194 | 1.367999705 | 0.007252537 | Up |
| CF003_0868 | bmgA | 1.4316771 | 0.011992058 | Up |
| CF003_1479 | traJ | 1.349081176 | 0.012173743 | Up |
| CF003_1199 | CF003_1199 | 2.811036824 | 0.014654938 | Up |
| CF003_1460 | CF003_1460 | 5.371049567 | 0.016317934 | Up |
| CF003_n06 | CF003_n06 | 1.350257963 | 0.027141994 | Up |
| CF003_0003 | wecH | 1.73661899 | 0.039014991 | Up |
| CF003_0874 | CF003_0874 | 1.309713516 | 0.039014991 | Up |
| CF003_1522 | menC | 1.56644774 | 0.042101453 | Up |
| CF003_n31 | hdoG | 1.061716866 | 0.04502463 | Up |

Table S3 Downregulated genes in *P. gingivalis* W83 Δ*trkA* strain

| Gene ID | Gene name | Log2FC | P value | Regulate |
| --- | --- | --- | --- | --- |
| CF003_1421 | fdx | -2.287215016 | 2.25E-61 | Down |
| CF003_1715 | cnaB | -1.661926995 | 8.91E-48 | Down |
| CF003_0306 | rnfG | -2.014827093 | 1.55E-41 | Down |
| CF003_1615 | frdA | -1.908875997 | 5.28E-40 | Down |
| CF003_1614 | frdB | -2.487360163 | 1.42E-36 | Down |
| CF003_1108 | terB | -1.737793625 | 3.01E-35 | Down |
| CF003_1547 | CF003_1547 | -2.278448974 | 1.70E-32 | Down |
| CF003_0307 | rnfE | -1.83463962 | 2.39E-31 | Down |
| CF003_1616 | sdhC | -1.62221776 | 3.08E-31 | Down |
| CF003_0303 | rsxB | -1.578325491 | 7.52E-31 | Down |
| CF003_0548 | nifJ | -1.455569434 | 9.65E-29 | Down |
| CF003_2117 | rpsP | -1.537817004 | 1.27E-24 | Down |
| CF003_1121 | asnS | -1.279130915 | 1.85E-24 | Down |
| CF003_1543 | yneP | -1.5310879 | 1.33E-23 | Down |
| CF003_1844 | kgp_4 | -1.238185403 | 7.48E-23 | Down |
| CF003_1545 | sodB | -1.667412696 | 1.34E-22 | Down |
| CF003_0547 | bioM | -1.468051783 | 4.15E-22 | Down |
| CF003_1144 | prfB | -1.232258922 | 5.45E-22 | Down |
| CF003_1089 | srrA | -1.206347363 | 7.47E-22 | Down |
| CF003_0195 | rbr | -1.670599092 | 1.12E-21 | Down |
| CF003_0144 | CF003_0144 | -1.628407778 | 1.14E-20 | Down |
| CF003_2034 | pyrK_2 | -1.462766806 | 6.42E-20 | Down |
| CF003_0304 | rnfC | -1.206449126 | 4.63E-19 | Down |
| CF003_0592 | rpmE | -1.363612717 | 5.91E-19 | Down |
| CF003_0332 | rho | -1.285500701 | 1.17E-18 | Down |
| CF003_0046 | cdsA | -1.077816322 | 2.25E-18 | Down |
| CF003_2216 | CF003_2216 | -1.301176079 | 2.82E-18 | Down |
| CF003_0377 | rpsB | -1.00374776 | 2.88E-18 | Down |
| CF003_0429 | korA_1 | -1.21685819 | 1.43E-17 | Down |
| CF003_0627 | rbp | -1.305245041 | 1.18E-16 | Down |
| CF003_1812 | korA_2 | -1.001081566 | 1.33E-16 | Down |
| CF003_1069 | kdd | -1.038683335 | 1.67E-16 | Down |
| CF003_2182 | nqrA | -1.103678101 | 4.48E-16 | Down |
| CF003_1117 | mdtK | -1.392307828 | 6.81E-16 | Down |
| CF003_1786 | CF003_1786 | -1.396244103 | 7.58E-16 | Down |
| CF003_1758 | rpsO | -1.575100792 | 7.59E-16 | Down |
| CF003_1122 | rluB | -1.046680649 | 1.23E-15 | Down |
| CF003_0302 | fis | -1.175745368 | 2.38E-15 | Down |
| CF003_0167 | rplY | -1.112395256 | 3.37E-15 | Down |
| CF003_0308 | rsxA | -1.542701726 | 4.66E-15 | Down |
| CF003_0006 | mepA_1 | -1.152095514 | 4.83E-15 | Down |
| CF003_2140 | rpmF | -1.771981525 | 5.48E-15 | Down |
| CF003_1013 | scpC | -1.069224207 | 5.52E-15 | Down |
| CF003_2177 | nqrF | -1.346198867 | 2.30E-14 | Down |
| CF003_1964 | wbaP_1 | -1.103389984 | 1.23E-13 | Down |
| CF003_0430 | korB_1 | -1.164311321 | 1.65E-13 | Down |
| CF003_2179 | nqrD | -1.601098077 | 2.61E-13 | Down |
| CF003_2033 | gltD | -1.029286269 | 6.58E-13 | Down |
| CF003_0435 | ptpZ | -1.127081262 | 6.91E-13 | Down |
| CF003_1640 | dinF | -1.233600108 | 8.00E-13 | Down |
| CF003_1540 | queA_1 | -1.092145471 | 1.11E-12 | Down |
| CF003_2213 | nasD | -1.225428218 | 2.36E-12 | Down |
| CF003_0712 | CF003_0712 | -1.201303828 | 2.39E-12 | Down |
| CF003_0375 | rplM | -1.019615757 | 2.94E-12 | Down |
| CF003_0745 | gloA | -1.056664245 | 7.66E-12 | Down |
| CF003_0141 | parB | -1.010148512 | 8.02E-12 | Down |
| CF003_1828 | pg-lp | -1.455514716 | 1.34E-11 | Down |
| CF003_0143 | ybeM | -1.133596909 | 3.17E-11 | Down |
| CF003_1960 | rpmB | -1.155661641 | 4.30E-11 | Down |
| CF003_2180 | nqrC | -1.21914184 | 6.48E-11 | Down |
| CF003_1393 | mrdA | -1.051144595 | 1.33E-10 | Down |
| CF003_0315 | rpmA | -1.115192425 | 1.35E-10 | Down |
| CF003_2178 | nqrE | -1.208897991 | 1.45E-10 | Down |
| CF003_0305 | rsxD | -1.024405374 | 2.83E-10 | Down |
| CF003_0286 | CF003_0286 | -1.34620683 | 3.13E-10 | Down |
| CF003_2139 | CF003_2139 | -1.044495896 | 3.53E-10 | Down |
| CF003_2126 | yggS | -1.047552419 | 7.20E-10 | Down |
| CF003_1959 | rpmG | -1.225307856 | 7.20E-10 | Down |
| CF003_0475 | hemN | -1.192458333 | 1.13E-09 | Down |
| CF003_1548 | prtT | -1.140872388 | 1.73E-09 | Down |
| CF003_0108 | epsD | -1.016566527 | 1.73E-09 | Down |
| CF003_1427 | speB | -1.040793511 | 1.76E-09 | Down |
| CF003_0018 | CF003_0018 | -1.211963683 | 3.00E-09 | Down |
| CF003_1723 | rpsT | -1.332135697 | 8.72E-09 | Down |
| CF003_0587 | yadS | -1.052452733 | 8.49E-08 | Down |
| CF003_0082 | ygjK_2 | -1.00056663 | 1.41E-07 | Down |
| CF003_1813 | psaC | -1.006714604 | 2.24E-07 | Down |
| CF003_0083 | CF003_0083 | -1.011923832 | 2.27E-07 | Down |
| CF003_2006 | CF003_2006 | -1.111534024 | 4.30E-07 | Down |
| CF003_0989 | rplT | -1.159762874 | 4.57E-07 | Down |
| CF003_1259 | nrdG | -1.018430726 | 5.23E-07 | Down |
| CF003_1098 | CF003_1098 | -1.01328164 | 1.54E-06 | Down |
| CF003_0385 | rpsU | -1.073137057 | 4.68E-06 | Down |
| CF003_n15 | pntA | -1.011141387 | 5.32E-06 | Down |
| CF003_1495 | topB_3 | -1.136609694 | 0.000283672 | Down |
| CF003_0656 | rpmH | -1.1252642 | 0.000555811 | Down |


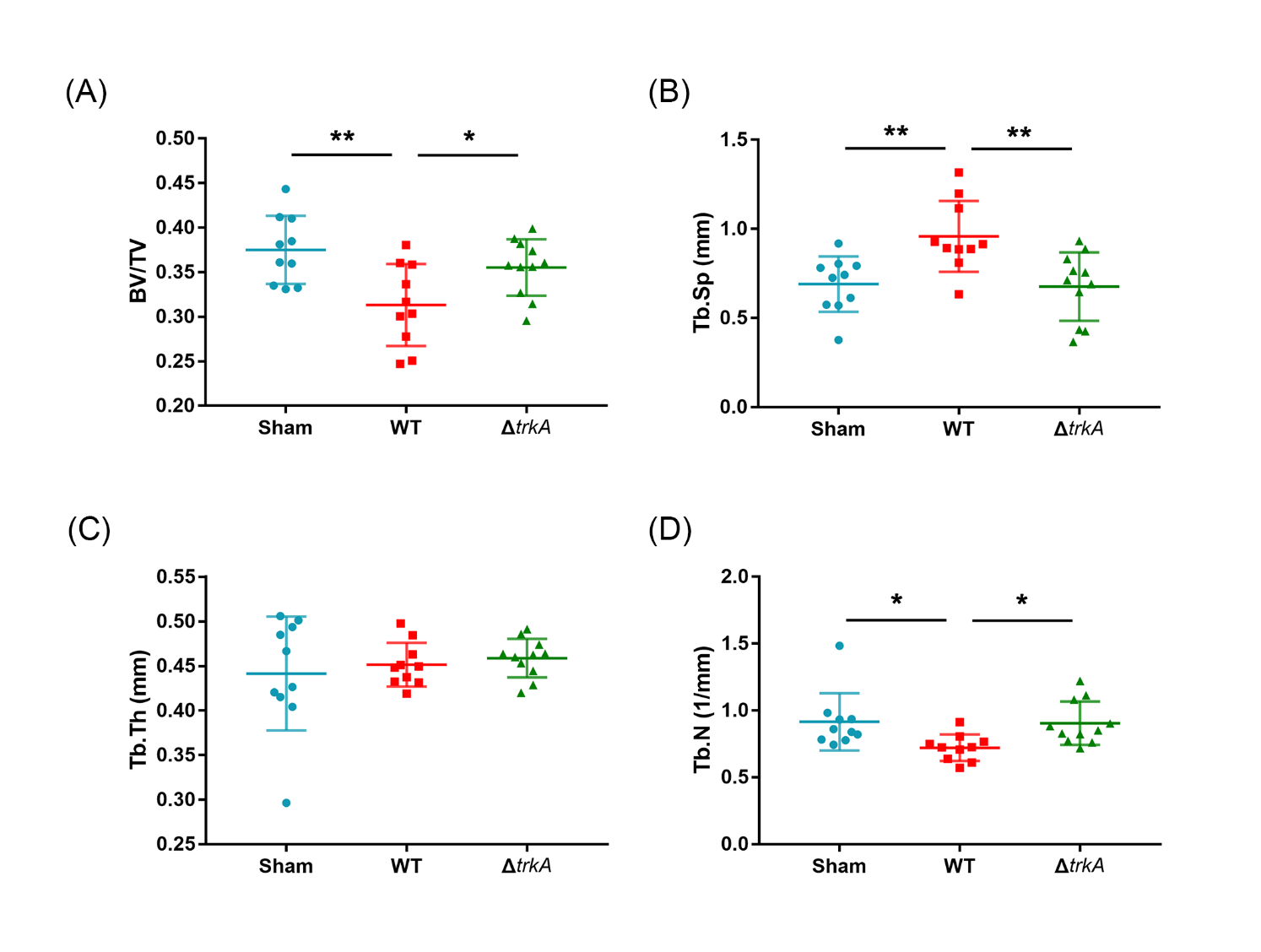


**Supplementary Figure 1.** Bone volume and density analysis of murine alveolar bone. (A) bone volume fraction, (B) trabecular separation, (C) trabecular thickness and (D) trabecular number of the three-dimensional micro-CT reconstruction were analyzed. All data are in means ± SD form. *p < 0.05, **p < 0.01.
